# Saddle Aortic Embolus With Paraplegia in a 60-Year-Old Diabetic and Hypertensive Patient: A Rare Case of Acute Lower Limb Ischemia

**DOI:** 10.7759/cureus.72362

**Published:** 2024-10-25

**Authors:** Nabil Al_Madhwahi, Aref Al-Hashedi, Mohammed A Alshujaa, Haitham M Jowah

**Affiliations:** 1 Department of Vascular Surgery, Faculty of Medicine and Health Science, Sana’a University, Sana'a, YEM; 2 Department of Vascular Surgery, Al-Thawra Modern General Hospital, Sana'a, YEM; 3 Department of Vascular Surgery, Faculty of Medicine and Health Science, Sana'a University, Sana'a, YEM; 4 Department of Surgery, Faculty of Medicine and Health Science, Sana'a University, Sana'a, YEM

**Keywords:** acute lower limb ischemia, case report, paraplegia, saddle aortic embolus, thrombectomy

## Abstract

Acute lower limb ischemia (ALI) is a vascular emergency that necessitates prompt intervention to avert irreversible damage. The relationship between ALI and paraplegia is a rare occurrence, particularly in patients with vascular risk factors such as diabetes mellitus (DM) and hypertension (HTN). We present the case of a 60-year-old male with a medical history of DM, HTN, and ischemic heart disease (IHD) who developed acute paraplegia 12 days after undergoing coronary artery bypass grafting (CABG). Initially suspected to have Guillain-Barré syndrome (GBS), further evaluation revealed occlusion of the abdominal aorta and iliac arteries. The patient underwent emergency bilateral fasciotomy and thrombectomy, resulting in partial recovery of lower limb function, with motor and sensory evaluations showing improvement in the right limb but limited recovery in the left. Postoperative complications included acute kidney injury, which was effectively managed. This case highlights the necessity of a broad differential diagnosis for rapidly progressing lower limb paralysis in patients with significant vascular risk factors. Timely diagnosis, prompt intervention, and careful monitoring of outcomes are essential for optimizing results in complex cases of ALI.

## Introduction

Acute lower limb ischemia (ALI) is a life-threatening vascular emergency characterized by a sudden reduction in blood flow to a limb. This can result in significant morbidity and potential limb loss if not treated promptly. The annual incidence of ALI is approximately 14 per 100,000 people, and it most commonly arises from thrombotic or embolic arterial occlusion [1]. The condition is often identified by the “six Ps”: pain, pallor, pulselessness, paresthesia, paralysis, and poikilothermia, which are the hallmarks of the clinical presentation [2].

In rare cases, paraplegia, an unusual manifestation that challenges the diagnostic process, can complicate ALI. Paraplegia, as a result of vascular insufficiency, is particularly uncommon in patients with underlying vascular risk factors such as diabetes mellitus (DM) and hypertension (HTN) [3]. These patients are more prone to thrombotic and embolic events due to systemic atherosclerosis and endothelial dysfunction [4]. Additionally, previous surgical interventions, such as coronary artery bypass grafting (CABG), further elevate the risk of embolization [5].

We present a rare case of a 60-year-old man who developed acute paraplegia following ALI. Initially misdiagnosed as Guillain-Barré syndrome (GBS), the case highlights the diagnostic challenges and underscores the importance of a multidisciplinary approach for timely diagnosis and intervention. To our knowledge, this is one of the few reported cases of saddle aortic embolism leading to paraplegia in a patient with multiple vascular risk factors.

This case report was posted as a preprint on the Research Square server on February 13, 2024.

## Case presentation

A 60-year-old male patient with a documented history of DM type II (DM II), HTN, and ischemic heart disease (IHD) was receiving regular treatment with oral antihyperglycemic, antihypertensive, and anti-ischemic medications. Twelve days post-CABG, he suddenly felt severe pain in his right lower limb that lasted for 30 minutes. The pain quickly got worse until he couldn’t move either lower limb and had lost all sensory function up to the inguinal region. At a private outpatient department (OPD) clinic, the patient sought medical consultation and received a preliminary diagnosis of GBS.

After this diagnosis, we initiated a diagnostic workup that included electromyography (EMG), nerve conduction studies (NCSs), and spinal computed tomography (CT). Sixteen hours after the onset of symptoms, cyanosis began to manifest in both feet. The Doppler ultrasound revealed blockages in the abdominal aorta, the common iliac artery (CIA), and the external iliac artery (EIA). There was also a tardus parvus flow from the common femoral artery (CFA) downward; the results showed that the EIA was about 90% blocked, and the flow rate in the CFA was less than 20 cm/s.

Our hospital received the patient 24 hours after the onset of symptoms. Upon arrival, he was conscious, oriented, and in stable condition, although he experienced slight dyspnea. Physical examination revealed mottling in both lower limbs, extending to the distal thigh on the right side and below the knee on the left side. Both lower limbs were cold, and pulses were absent from both CFAs. After administering 5000 IU of intravenous heparin as an immediate intervention, the patient underwent surgery 26 hours after the onset of symptoms.

We performed a bilateral leg fasciotomy, which revealed dusky-colored muscles with minimal dark bleeding. Upon electrocautery, we observed weak muscle contractions. Upon exploring both CFAs, we extracted multiple large thrombi from the proximal and distal regions (Videos 1, 2).

**Video 1:** Surgical extraction of large arterial thrombus from the right common femoral artery (CFA)

**Video 2:** Surgical extraction of large arterial thrombus from the left common femoral artery (CFA)

We performed arterial repair using polypropylene 6-0 sutures and closed the wound after placing a vacuum drain. We then transferred the patient to the intensive care unit (ICU) for postoperative observation and medical management. We found the lower limb pulse palpable up to the level of both popliteal arteries (PTAs).

After the arterial repair, we monitored the patient in the ICU for 48 hours. During this time, he developed postoperative complications, including elevated creatinine levels of 3.2 mg/dL and potassium levels of 5.2 mmol/L, which indicated potential acute kidney injury and hyperkalemia. We managed these complications with careful fluid management and electrolyte monitoring, ultimately normalizing his creatinine and potassium levels within 24 hours. Despite regaining movement in his right lower limb after 12 hours, the left lower limb showed no improvement. After six days, the patient was discharged in stable condition, with a good pulse detected in both lower limbs. While motor and sensory functions returned intact in the right lower limb, the left lower limb exhibited only slight motor function in the thigh (grade 2), and sensation was restricted to painful stimuli from the mid-thigh.

## Discussion

A 60-year-old male patient with a history of DM, HTN, and IHD presented with sudden severe lower limb pain and subsequent paralysis following CABG. Initially diagnosed with GBS, further investigation revealed arterial thrombosis occluded the abdominal aorta and iliac arteries. Prompt administration of heparin was followed by surgical intervention, including bilateral leg fasciotomy and thrombus extraction. While the right lower limb improved, the left lower limb experienced limited recovery. The strengths of this approach include prompt recognition and timely intervention upon patient arrival at the hospital. However, the limitations of this approach include misdiagnosis at the initial presentation, delayed hospital presentation, and unilateral motor function improvement.

The case presented herein, involving a patient with saddle aortic emboli and paraplegia, represents a rare occurrence of ALI, which is notable for the rapid progression of symptoms and initial misdiagnosis. The patient’s history of DM, HTN, IHD, and recent CABG placed him at an increased risk of vascular events. The sudden onset of severe lower-limb pain progressing to paralysis in both lower limbs aligns with the classic presentation of ALI, which is often described by the “six Ps”: pain, pallor, pulselessness, paresthesia, paralysis, and poikilothermia.

The initial provisional diagnosis of GBS, a rare neurological disorder in which the body’s immune system mistakenly attacks part of its peripheral nervous system, illustrates the challenges in diagnosing rare presentations of more common conditions. Although GBS patients may present with rapidly progressing limb weakness, the sudden onset and severe pain experienced by these patients are more characteristic of vascular events than neurological events [2].

Given the patient’s symptoms and medical history, the differential diagnosis should include ALI. Delays in the correct diagnosis allow ischemia to progress, leading to cyanosis and mottling, which indicate severe tissue hypoxia and imminent necrosis. This delay may have contributed to poorer recovery of the patient’s left lower limb [3].

Occlusion of both the CIA and EIA indicates a saddle embolus, a large embolus that straddles the bifurcation of the artery. In this case, the embolus likely originated from the heart and was possibly related to the patient’s recent CABG and DM history [4-6]. The thrombi removed from the patient’s CFAs were likely secondary emboli that further occluded the distal circulation.

Immediate anticoagulation with heparin followed by surgical intervention, including fasciotomy and thrombectomy, is necessary to prevent irreversible tissue damage and possible limb loss. Despite these interventions, the patient’s recovery was complicated by acute kidney injury, possibly due to emboli occlusion in the renal arteries or a systemic inflammatory response to acute ischemia. Moreover, acute kidney injury is a risk factor for cardiac catheterization and elective coronary artery bypass surgery [7].

Partial recovery of motor and sensory function in the left lower limb indicated the severity of the ischemic injury. The fact that sensation was limited to painful stimuli from the mid-thigh suggested that nerve function was more severely affected in the distal versus proximal portions of the limb, consistent with the ischemic insult being more severe distally due to CFA occlusion.

The mechanism underlying the paraplegia observed in this patient can be attributed to the occlusion of the lumbar and sacral arteries, which originate from the abdominal aorta and provide blood to the spinal cord. As described in a case report by Olearchyk (2018) [8], a saddle embolus lodged at the level of L4 can effectively cut off the blood supply to the lower spinal cord, resulting in acute ischemia and subsequent paraplegia.

Another case report by Chandrashekar et al. (2019) described a patient who experienced sudden paraplegia onset due to a saddle embolus [9]. The patient’s paraplegia resolved over two months.

The pathophysiology of ALI involves complex cellular and molecular mechanisms. When arterial blood flow is abruptly removed, as in the case of a saddle embolus, affected tissues are subjected to ischemia, leading to hypoxia. This phenomenon triggers a cascade of events, including reactive oxygen species production, inflammatory pathway activation, and cell death. If ischemia is not promptly reversed, irreversible tissue damage and necrosis can occur, leading to clinical manifestations of ALI, including severe pain, pallor, pulselessness, and paralysis [10].

Paraplegia can also be attributed to a reduction in perfusion pressure, as observed in hypotensive infarction of the spinal cord [11]. The documented correlation between acute aortic occlusion and paraplegia underscores the critical importance of recognizing and addressing such acute ischemic events to prevent devastating neurological complications.

Successful management of ALI, which involves intravenous heparin administration, bilateral leg fasciotomy, and thrombectomy, is consistent with the standard approach for treating ALI [11]. However, the development of residual motor and sensory deficits in the left lower limb underscores the importance of early intervention for preventing limb loss and improving overall outcomes in patients with ALI.

## Conclusions

This case underscores the necessity of maintaining a broad differential diagnosis for rapidly progressing lower limb paralysis, particularly in patients with vascular risk factors such as diabetes and HTN. Timely diagnosis and intervention for ALI are crucial for improving patient outcomes. Clinicians should be vigilant in recognizing the classic signs of ALI, utilize rapid diagnostic tools like Doppler ultrasound for prompt identification of arterial occlusions, and adopt a multidisciplinary approach involving vascular surgeons, interventional radiologists, critical care specialists, and neurologists. Additionally, continuous monitoring for postoperative complications, including acute kidney injury, is essential for facilitating early intervention and optimizing patient care. Implementing these strategies can enhance the management of ALI and prevent adverse complications.
